# Effect of modified clivoaxial angle on surgical decision making and treatment outcomes in patients with Chiari malformation type 1

**DOI:** 10.3389/fsurg.2023.1143086

**Published:** 2023-05-05

**Authors:** Mehmet Emin Akyuz, Mehmet Kürşat Karadag, Mehmet Hakan Sahin

**Affiliations:** Neurosurgery Depertmant, School of Medicine, Ataturk University, Erzurum, Türkiye

**Keywords:** Chiari malformation type 1, ventral compression, Chicago Chiari outcome scale, modified clivoaxial angle, surgical outcome, radiological criteria, neurosurgery

## Abstract

**Introduction:**

Chiari malformation type 1 (CM1), a complex pathological developmental disorder of the craniovertebral junction, is typically characterized by herniation of the cerebellar tonsils from the foramen magnum. Treatment using posterior fossa decompression alone without taking the ventral cervico-medullary compression into consideration may lead to unsatisfactory treatment outcomes. The current study evaluated the utility of the modified clivoaxial angle (MCAA) in assessing ventral compression and also examined its effect on treatment outcomes.

**Method:**

This retrospective study included 215 adult patients who underwent surgical treatment for CM1 at one medical center over a 10-year period. The following surgical techniques were used to decompress the posterior fossa: (a) PFD: bone removal only; (b) PFDwD: bone removal with duraplasty; and (c) CTR: cerebellar tonsil resection. The morphometric measurements of the craniovertebral junction (including MCAA) were recorded using preoperative images, and the postoperative clinical status was evaluated using the Chicago Chiari outcome scale (CCOS).

**Results:**

MCAA was positively correlated with the CCOS score and also independently predicted treatment outcome. To enable Receiver operating characteristic (ROC) curve analysis of CCOS scores, the patients were divided into three groups based on the MCAA cut-off values, as follows: (a) severe (*n* = 43): MCAA ≤ 126; (b) moderate (*n* = 86): 126 < MCAA ≤ 138; and (c) mild (*n* = 86): MCAA > 138. Group a exhibited severe ventral cervico-medullary compression (VCMC), and their CCOS scores for the PFD, PFDwD, and CTR groups were 11.01 ± 1.2, 11.24 ± 1.3, and 13.01 ± 1.2, respectively (*p* < 0.05). The CCOS scores increased with widening of the MCAA angle in all surgical groups (*p* < 0.05). Furthermore, patients with mild MCAA (>138°) exhibited 78% regression of syringomyelia, and this was significantly greater than that observed in the other groups.

**Discussion:**

MCAA can be used in the selection of appropriate surgical techniques and prediction of treatment outcomes, highlighting the importance of preoperative evaluation of ventral clivoaxial compression in patients with CM1.

## Introduction

Chiari malformation type 1 (CM1), defined as herniation of the cerebellar tonsils >3–5 mm from the foramen magnum with or without the presence of syringomyelia (1, 2), can be diagnosed radiologically and affects approximately 1% of the general population (3). Associated neurological symptoms are often vague or non-specific and include headaches, ocular and autoneurological disturbances, lower cranial nerve symptoms, cerebellar ataxia, and spasticity (4, 5). Symptomatic CM1 is more prevalent among adults (affecting approximately 60% of the population) compared to children (4).

Surgical treatment of isolated CM1 typically involves removal of the brainstem compression and expansion of the posterior fossa volüme (6). It is usually recommended in patient exhibiting cough headaches or abnormal auditory/cerebellar/bulbar findings upon neurological examination (7). However, there is considerable debate around the suitability of various surgical approaches, with decompression of the posterior fossa using craniectomy (with or without dural opening) and removal of the posterior arch of the C1 vertebra being the most popular choice because of the short surgical duration and relatively low risk (8). The advantages and disadvantages of the three key surgical techniques have been summarized in Table 1 (9).

**Table 1:** Comparison of three surgical techniques, table created based on information from “Diagnosis and treatment of Chiari malformation and syringomyelia in adults: international consensus document, 2019, Milan”.

Surgical Technique	Advantages	Disadvantages
Only bone removed	Shorter surgical time, decrased blood loss, shorter hospital stay, lower risk of complications	Possibility of inadequate decompression; limited regression of syringomyelia or re-accumulation of syringomyelia due to failure to restore CSF flow; risk of recurrence of symptoms.
Bone removal with duraplasty	Greater opportunity for decompression; increased posterior fossa volume, thus allowing more CSF flow.	Risk of CSF leakage and related complications (infection, wound site problems, etc.); formation of arachnoidal scar tissue; foreign body reaction or inflammatory complications associated with grafts used during duraplasty.
Bone removal and cerebellar tonsil resection	Possibility of complete alleviation of brain stem compression; minimum risk of symptom recurrence.	Prolonged surgical duration; increased risk of injury to neurovascular structures; complications of duraplasty

Although the pathophysiology of CM1 is not fully understood, it is generally accepted that crowding at the cervicomedullary junction causes symptoms as a result of pressure on surrounding tissues and disruption of CSF flow (10). It has been shown in studies that not only the herniated cerebellar tonsils but also the soft tissues in the retro-odontoidal region (transverse ligament and tectorial membrane) may create pressure on this junction and affect the clinic (11). We think that it may be wrong to determine the surgical decompression limits of patients without taking this soft tissue bulge into account. The effect of the modified clivoaxial angle, which evaluates both the skull base angles and retroodontoidal soft tissues together at the cervicomedullary junction, on the clinical outcome of the patients and on postoperative recovery has been shown by studies (12), and we think that it is an appropriate radiologic criterion for evaluating the ventral cervicomedullary crowding.

Although there are numerous age- and gender-matched radiological studies focusing on the treatment of CM1, few have explored clinical treatment outcomes to date. Therefore, the current study used preoperative radiological images of patients undergoing posterior decompression for CM1 to evaluate the utility of the modified clivoaxial angle (MCAA) in assessing ventral compression and to also examine its effect on treatment outcomes.

## Materials and method

This retrospective study was approved by the Ataturk University local ethics committee (protocol number: B.30.2.ATA.0.01.00/662) and included 215 patients diagnosed with CM1 who underwent surgical treatment at our clinic between 2010 and 2020. CM1 diagnosis was confirmed when magnetic resonance imaging (MRI) showed that the cerebellar tonsils had descending >5 mm from the foramen magnum. Preoperative images were used to confirm absence of basilar invagination and atlantoaxial dislocation. Adult patients (>18) who underwent surgery and whose all information was available during follow-up were included in the study. Patients with tumors, trauma, and vascular diseases at the cervical region were excluded and, none of them had basilar invagination or atlanto-axial dislocation, as confirmed on roentgenogram or computed tomography of the craniovertebral junction.

## Surgical techniques

The patient was placed in the concorde position on the operating table and their head was stabilized. A classical suboccipital incision was made under general anesthesia and a 2 × 2 cm bony window was created using occipital craniectomy to allow removal of the C1 posterior arch (PFD). Our criteria for decompression were as follows; it should be wide enough over the foramen but not too wide to prevent cerebellar slippage, always include C1 laminectomy but never extend to C2 because of the risk of CVJ instability. Among patients who underwent duraplasty, a U-shaped incision was made in the dura mater and expanded using a synthetic dural patch (PFDwD). Among those who underwent tonsillar resection, the cerebellar tonsils were coagulated or resected from the tip until the foramen of Magendie was visible and flow was assured (CTR). Water-tight closure of the surgical site was carried out thereafter. All surgical procedures were performed by a surgical team under the supervision of a senior surgeon.

## Preoperative radiological evaluation

**Figure 1:**
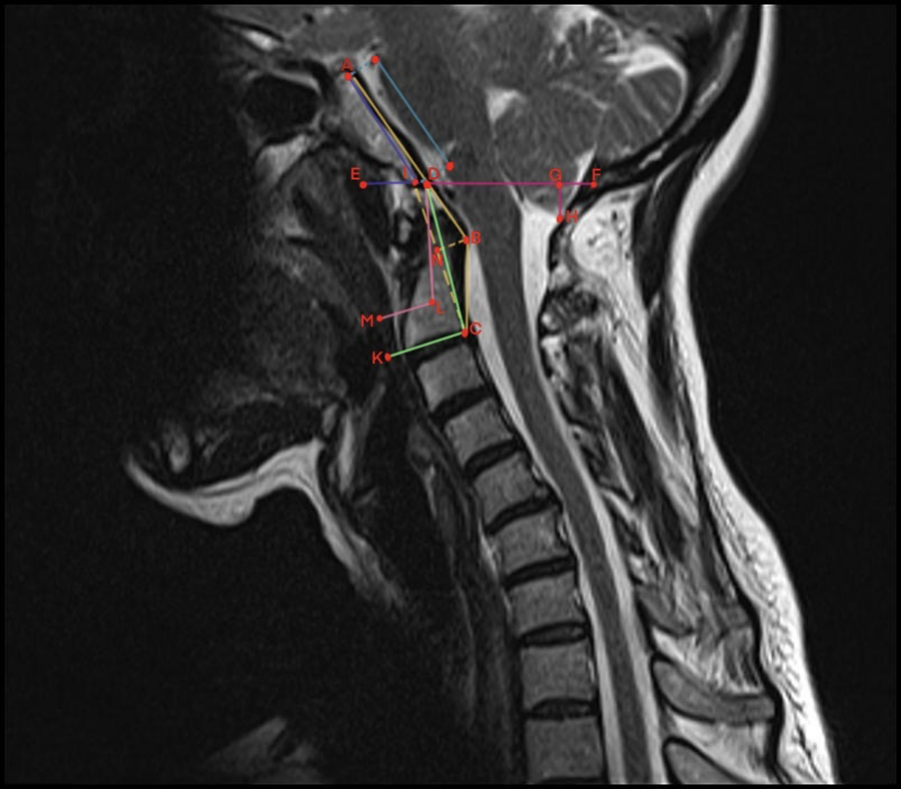
Schematization of craniovertebral morphometric measurements on sagittal T-2-weighted imaging. ABC, modified clivoaxial angle; GH, herniated cerebellar tonsil length; AD, clivus length; AIE, clivus angle; DCK, odontoid retroversion; DLM, odontoid retroflexion; DF, McRae line.

Preoperative evaluation of the patient was carried out using MRI. The patient's neck was kept in a neutral position and the following parameters were measured. The parameters measured in the preoperative MRI were as follows and a schematic drawing of these radiologic criteria is shown in Figure 1;

1. The length of the cerebellar tonsil extension below the foramen magnum. (GH)
2. Clivus length (the distance between the dorsum sellae and basion) (AD)
3. Clivus angle (angle between a line drawn along the clivus and the McRae line). (AIE)
4. Basion to C2 line distance (pB-C2; perpendicular distance between the ventral dura and a line joining the basion to the posterior portion of the axis body inferior endplate).
5. Cranial base angle (angle between a line running along the anterior cranial fossa to the tip of the dorsum sella and a line drawn along the posterior edge of the clivus).
6. McRae line (extending from the basion to opisthion).
7. Modified clivoaxial angle (MCAA; the angle between a line running along the clivus and a line tangent to the base of the C2 vertebra on the retro-odontoid soft tissue) (13). (ABC)
8. Odontoid retroversion (angle between the base of C2 and the intersection of a line from the odontoid tip). (DCK)
9. Odontoid retroflexion (the angle formed between the intersection of a line drawn from the odontoid synchondrosis and a line drawn from the odontoid tip). (DLM)
10. Syringomyelia (SM; evaluated as present/absent on preoperative MRI and shrinkage +/− on postoperative MRI).

## Outcome measures

Postoperative clinical evaluation was carried out 24 months after surgery using the Chicago Chiari outcome scale (CCOS) which includes pain and non-pain symptoms, functionality, and complications. The outcomes were categorized into improved (scores 13–16), unchanged (scores 9–12), or worsened (scores 4–8) (14) (Table 2).

**Table 2:** Table from Aliaga L, Hekman KE, Yassari R, et al. A novel scoring system for assessing Chiari malformation type I treatment outcomes. Neurosurgery. 2012;70(3):656–665 (14).

Chicago Chiari outcome scale				
Pain	Non-Pain	Functionality	Complications	Total Score
1-Worse	1-Worse	1-Unable to attend	1-ersistent complication, poorly controlled	4-Incapacitated outcome
2-Unchanged and refractoy medication	2-Unchanged or improved but impaired	2-Moderate impairment (<50% attendance)	2-Persistent complication, well controlled	9-Impaired outcome
3-Improved or controlled with medication	3-Improved and unimpaired	3-Mild impairment (>50% attendance)	4-Transient complication	12-Functionally outcome
4-Resolved	4-Resolved	4-Fully functional	4-Uncomplicated course	16-Excellent outcome

## Statistical analysis

All statistical analyses were performed using a statistical software (SPSS Statistics, version 22, IBM, Armonk, NY). Bivariate analyses (Pearson's and Spearman's correlation analysis) and multiple linear regression models were used to examine the association between preoperative MCAA and other MRI parameters. Bivariate analysis was also used to examine the association between MCAA and postoperative CCOS scores independent of the surgical method used. Receiver operating characteristic (ROC) curves were used to test the accuracy of MCAA in predicting CCOS scores and identifying the optimal threshold values. One-way analysis of variance was used to compare the CCOS scores of each surgical method by MCAA grade.

## Results

The study included 215 patients who underwent surgical treatment for CM1, of which 102 were female and 113 were male. The mean patient age, follow-up duration, and length of herniation of the cerebellar tonsil from the foramen magnum were 42 ± 4 years, 44 ± 8 months, and 10.8 + 4.2 mm, respectively.

**Figure 2:**
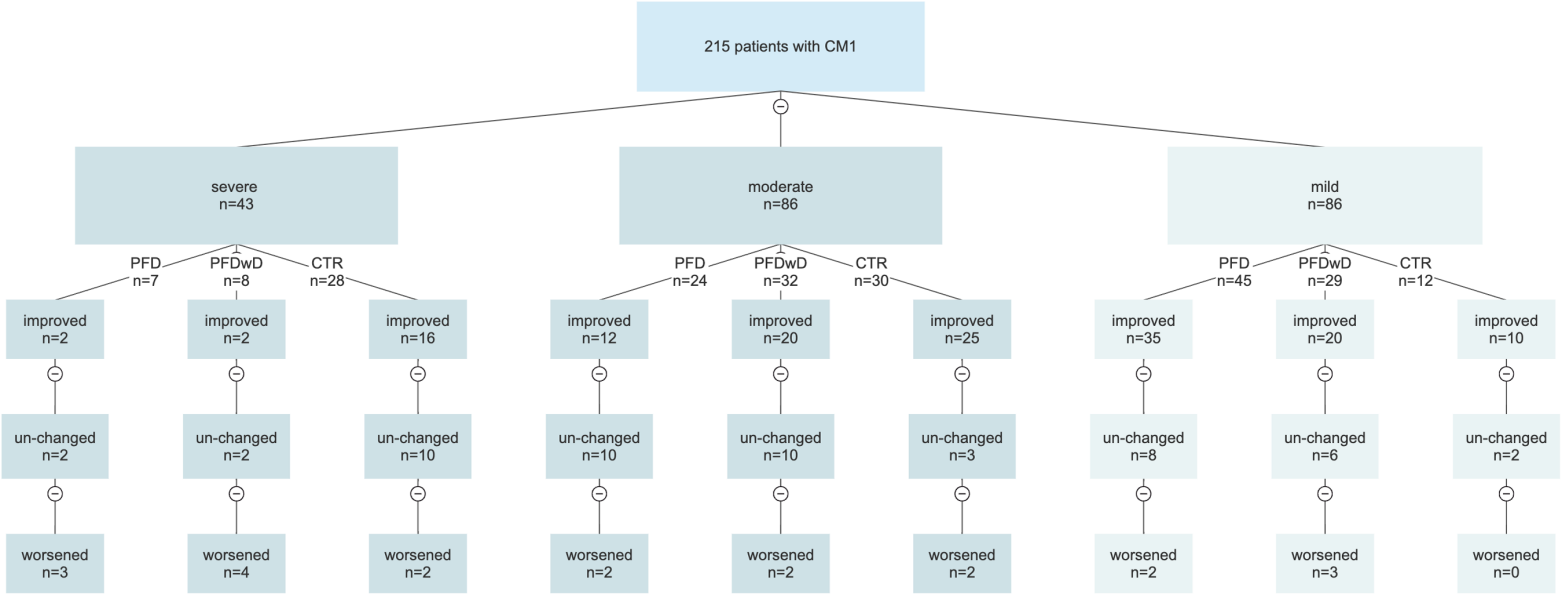
Flow chart showing the relationship between surgical technique and MCAA. Patients were divided into 3 groups according to MCAA determined by ROC analysis with CCOS scores; A = severe (MCAA ≤ 126), moderate (126 < MCAA ≤ 138) and mild (MCAA > 138).

The CCOS scores (mean value: 12.64 ± 2.1) were improved in 129 (60%) patients, unchanged in 75 patients (34.8%), and worsened in 11 patients (5.2%). Moreover, scores were seen to improve in 52% (33/64) of the PFD group (mean score: 12.24 ± 2.3), 64% (38/60) of the PFDwD group (mean score: 13.1 ± 1.2), and 73% (66/91) of the CTR group (mean score: 14.1 ± 1.8). A statistically significant difference in outcome scores was observed between the groups (*p* < 0.05). The flow-chart showing the CCOS scores of the patients according to the surgical technique performed is shown in Figure 2.

The list of surgical complications and medical complications diagnosed within 90 days after CM-1 surgery is presented in Table 3. The most common surgical complications were meningitis and wound site complications, which were more frequent in patients undergoing CTR. The number of reoperations due to complications was 5, 4 of which were dural graft failure and 1 wound repair.

**Table 3:** The list of complications seen within 90 days after surgery.

Complication	PFD 76 (*n*/%)	PFDwD 69 (*n*/%)	CTR 70 (*n*/%)
Meningitis	0/0	3/4.3	4/5.7
Wound infection	2/2.6	3/4.3	4/5.7
Dural greft complication	0/0	4/5.7	6/8.5
İatrogenic cerebrovascular infarction	0/0	0/0	1/1.4
Pulmonary complication/pneumonia	0/0	1/1.4	0/0
Urinary-renal complication	1/1.3	0/0	0/0
Thrombotic complication	0/0	0/0	1/1.4

**Table 4:** Correlation of MCAA with other parameters by bivariate analysis, *p* < 0.05 is statistically significance.

Variable	Value	*ρ*/r	*p* value
MCAA°	134.33 ± 10.27	1.00	NA
pB-C2 distance (mm)	8.63 ± 1.15	−0.47	0.00
Cranial base angle (°)	122.42 ± 7.41	−0.78	0.00
Clivus lenght (mm)	43.03 ± 2.46	0.39	0.00
Clivus angle (°)	45.24 ± 8.32	0.46	1.37
Tonsillar herniation (mm)	12.31 ± 5.20	−0.15	0.03
Odontoid retroversion (°)	93.08 ± 5.63	−0.32	0.00
Odontoid retroflexion (°)	108.52 ± 8.10	−0.29	0.00
McRae line (mm)	34.48 ± 3.70	−0.05	0.38
Illness duration (month)	44.46 ± 79.85	0.07	0.37
Operation (PFD/PFDwD/CTR)	76/69/70	0.08	0.37
BMI (kg/m^2^)	28.20 ± 4.07	0.01	0.79
Age (years)	44.77 ± 10.37	0.07	0.29
Sex (female/male)	102/113	0.02	0.72

NA, not applicable; ρ/r, correlation coefficient.

Values specified as bold are statistically significant.

The bivariate analysis showed a significant correlation between MCAA and clivus length, clivus angle, odontoid retroflexion, odontoid retroversion, pB-C2 distance, tonsillar herniation, and cranial basal angle (*p* < 0.05; Table 4). Multiple linear regression analysis showed that the clivus angle (+), pB-C2 distance (–), cranial basal angle (–), and odontoid retroversion (–) were independently correlated (*p* < 0.05; Table 5).

**Table 5:** Correlation of MCAA with other parameters by multiple linear regression model, *p* < 0.05 is statistically significance.

Variable	B	Beta	*p*	95% CI	
				Lower	Upper
Constant	**224**.**52**	**NA**	**0**.**00**	**202**.**54**	**236**.**41**
pB-C2 distance (mm)	**−1**.**82**	**−0**.**33**	**0**.**00**	**−2**.**42**	**−1**.**41**
Cranial base angle (°)	**−0**.**41**	**−0**.**51**	**0**.**00**	**−0**.**52**	**−0**.**44**
Clivus length (mm)	0.21	0.05	0.26	**−**0.04	0.30
Tonsillar Herniation (mm)	0.03	0.02	0.58	**−**0.11	0.17
Clivus angle (°)	**0**.**27**	**0**.**35**	**0**.**00**	**0**.**10**	**0**.**48**
Odontoid retroversion (°)	**−0**.**18**	**0**.**06**	**0**.**02**	**−0**.**49**	**−0**.**02**
Odontoid retroflexion (°)	**−**0.07	**−**0.04	0.16	**−**0.31	0.04

B, partial regression coefficient; Beta, standardized partial regression coefficient, NA, not applicable; CI, confidence interval.

Values specified as bold are statistically significant.

Bivariate analysis showed that the postoperative CCOS scores were significantly associated with preoperative MCAA, clivus angle, pB-C2 distance, cranial base angle, and odontoid retroflexion (*p* < 0.05; Table 6), while MCAA was independently correlated according to multiple linear regression analysis under the dummy variables of surgery type (*p* < 0.05) (Table 7).

**Table 6:** The bivariate analysis of MCAA and other craniovertebral junction parameters with CCOS.

Variable	Value	r	*p*
CCOS	12.64 ± 2.1	1	NA
MCAA (°)	134.33 ± 10.27	**0**.**52**	**0**.**00**
Clivus angle (°)	**45.24 **±** 8.32**	**0**.**17**	**0**.**00**
pB-C2 distance (mm)	**8.63 **±** 1.15**	**−0**.**27**	**0**.**00**
Cranial base angle (°)	**122.42 **±** 7.41**	**−0**.**29**	**0**.**00**
Clivus length (mm)	**43.03 **±** 2.46**	**0**.**27**	**0**.**00**
Tonsillar herniation (mm)	12.31 ± 5.20	**−**0.14	0.12
Odontoid retroversion (°)	93.08 ± 5.63	**−**0.89	0.26
Odontoid retroflexion (°)	**108.52 **±** 8.10**	**−0**.**16**	**0**.**03**

Values specified as bold are statistically significant.

**Table 7:** The multiple linear regression modal of MCAA and other craniovertebral junction parameters with CCOS (operations used as dumb variable).

Variable	B	Beta	*p*	95% CI	
				Lower	Upper
MCAA (°)	**0**.**07**	**0**.**83**	**0**.**00**	**0**.**04**	**0**.**95**
Clivus angle (°)	**−**0.02	**−**0.04	0.47	**−**0.07	0.01
pB-C2 distance (mm)	0.04	0.03	0.22	**−**0.04	0. 19
Cranial base angle (°)	0.02	0.21	0.26	**−**0.01	0.03
Clivus length (mm)	0.03	0.17	0.15	**−**0.01	0.08
Odontoid retroflexion (°)	**−**0.01	**−**0.06	0.47	**−**0.04	0.02
Operation1	**0**.**75**	**0**.**02**	**2**.**08**	**0**.**00**	**0**.**27**
Operation2	**1**.**00**	**0**.**04**	**2**.**65**	**0**.**00**	**0**.**65**

B, partial regression coefficient; Beta, standardized partial regression coefficient; CI, confidence interval.

Values specified as bold are statistically significant.

**Figure 3:**
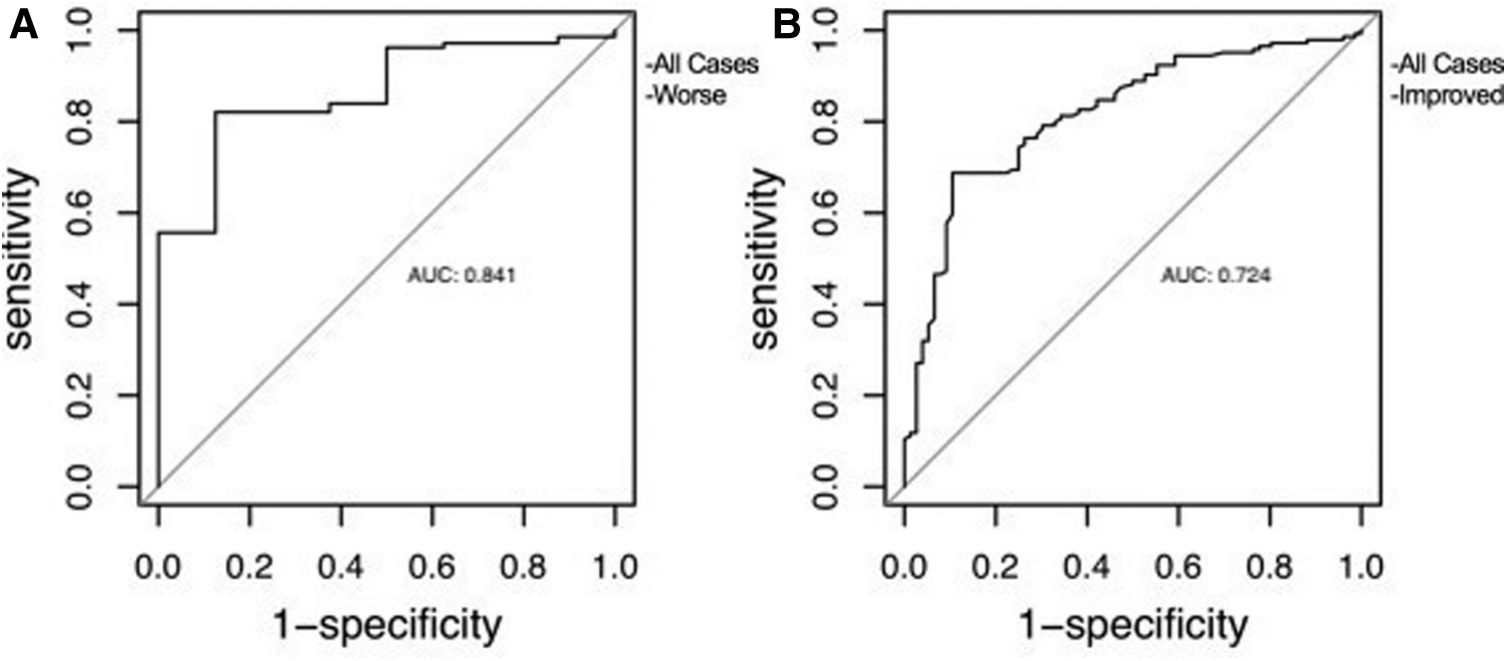
The receiver operating characteristic curve of MCAA used to predict clinical outcome. (A) for patients with worsening clinical status, (B) for patients improving clinical status.

The ROC curves showed that MCAA effectively predicted CCOS, with the area under the curve being 0.72 (*p* < 0.01) and 0.84 (*p* < 0.01) for better and poorer outcomes, respectively. Furthermore, as per Youden's index, the optimal MCAA threshold value for better and poorer outcomes was 138.12 and 126.25, respectively (Figure 3).

Preoperative ventral cervico-medullary compression was assessed by dividing the patients into three groups based on the optimal MCAA threshold value, as follows: (a) severe (*n* = 43): MCAA ≤ 126; (b) moderate (*n* = 86): 126 < MCAA ≤ 138; and (c) mild (*n* = 86): MCAA > 138. A statistically significant difference in mean CCOS scores was observed between the three groups (severe: 11.69 ± 1.2, moderate: 12.85 ± 1.4, mild: 13.61 ± 1.7), with group c exhibiting the greatest improvement (Table 8).

**Table 8:** Mean CCOS scores and percentages of change of patients grouped by MCAA scores.

	Group A	Group B	Group C	*p* value
Mean CCOS score	11.69 ± 1.2	12.85 ± 1.4	13.61 ± 1.7	<0.01
Improved (*n*/%)	20/53.4%	38/66.2%	65/75.5%	<0.01
Unchanged (*n*/%)	22/32.5%	46/26.7%	10/18.6%	<0.01
Worsed (*n*/%)	9/20.9%	6/6.9%	5/5.8	<0.01

**Figure 4:**
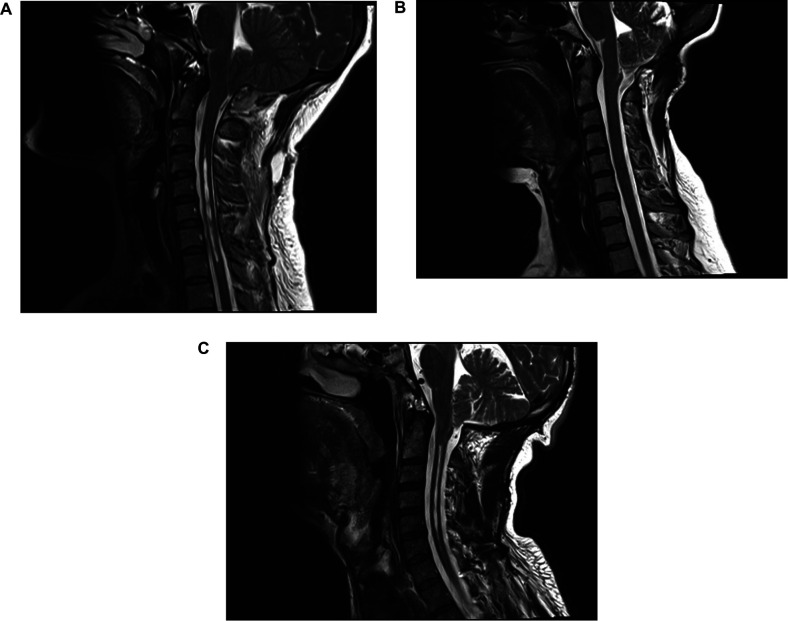
(**A**) Preoperative sagittal T-2 weighted MRI image of a patient with CM1 with syringomyelia, (**B**) MRI image taken at the 6th month follow-up after suboccipital craniectomy shows that the syringomyelia is not shrinkage and the crowding at the craniovertebral junction continues, (**C**) after CTR, the syringomyelia is shrinkage and the CVJ is decompressed.

Table 9 shows patient characteristics by the surgical technique used. In the current study, 120 patients presented with preoperative SM, of which 72 exhibited post-surgical regression. Moreover, group c exhibited the highest shrinkage (78%) regardless of surgical technique use. In addition, according to bivariate logistic regression analysis, the most frequent syringomyelia shrinkage in the group with severe MCAA was observed in the CTR group (*p* < 0.05). MRI images of a patient with CM1 with syringomyelia preoperatively, after PFD and after CTR are shown in Figure 4.

**Table 9:** Variation of CCOS scores between groups after 3 different surgical approaches.

MCAA groups	CCOS score			*p* value
	PFD	PFDwD	CTR	
Group A	11.01 ± 1.2	11.24 ± 1.3	13.01 ± 1.2	0.03
Group B	11.24 ± 1.4	12.14 ± 1.5	13.45 ± 1.3	<0.01
Group C	13.12 ± 1.3	13.96 ± 1.6	13.98 ± 1.5	0.04

## Discussion

Chiari malformations typically affect the hindbrain, with CM1 representing various degrees of herniation of the cerebellar tonsils through the foramen magnum (15). Based on the anatomical position of the tonsillar herniation, the clinical presentation of CM1 may include brainstem, cerebellar, or spinal cord anomalies (16). Although craniectomy with or without duraplasty (posterior decompression) continues to be the treatment of choice, there is limited consensus in the literature regarding its efficacy (17). For example, a recent report suggested that posterior decompression alone may be insufficient due to the complex nature of the pathology affecting the craniovertebral junction and the various morphometric parameters that play a role in clinical outcomes (18).

Osseous anomalies such as basilar invagination, retroflexed odontoid process, and platybasia associated with CM1 can compress the herniated cerebellar tonsils in the foramen magnum, contributing to the development of hydrocephalus and syringomyelia and, consequently, affecting clinical outcomes after posterior decompression (19, 20). The soft tissues, transverse ligaments, and tectorial membrane in the retro-odontoid space can also contribute to compression of the craniovertebral junction. Oldfield et al., suggested that continuous dynamic piston-like movements of the cerebellar tonsils at the craniovertebral junction can inhibit CSF flow and lead to soft tissue hypertrophy and further compression (21). To date, studies evaluating ventral compression of the craniovertebral junction have either focused on bony pathologies only or examined the soft tissues. However, we hypothesized that the MCAA, which takes both parameters into consideration, can provide a clearer understanding of the underlying pathological situation.

Brockmeyer et al. reported the importance of craniocervical angles as a cause of failure after suboccipital craniectomy in children treated for CM1 (22). In this study, they used the definition of “Complex Chiari” for patients with one or more of the following radiological criteria with herniation of the cerebellar tonsils through the foramen magnum; medullary kink, retroflexed odontoid, abnormal clival-cervical angle, occipitalization of the atlas, basilar invagination, syringomyelia or scoliosis. Dr. Brockmeyer et al. recommended anterior decompression for patients with pB-C2 distances >5 mm and bulbar symptoms (22). In our study, we tried to emphasize the importance of ventral compression in parallel with the study mentioned by this team, but differently, we concluded that patients who do not have enough ventral compression to need anterior decompression but need wide resection (CTR).

The findings of the current study showed that MCAA was associated with numerous factors affecting ventral compression, including pB-C2 distance and odontoid retroflexion. Chibbaro et al. in their study, they showed how useful anterior decompression is for clinical recovery in the treatment of pathological conditions that cause ventral compression (23). Fernandese et al., previously carried out 3-D volumetric measurement of the posterior fossa and found that it exhibited a correlation with the cranial basal angle (24). Although the current study did not measure the posterior fossa, a negative correlation was observed between the cranial basal angle and MCAA. This suggests that the latter indirectly reflects the volume of the posterior fossa, with a reduced MCAA indicating a smaller posterior fossa and, consequently, greater congestion. The clivus angle also plays a critical role in the width of the foramen magnum which, when narrowed, can lead to further blockage of CSF flow. The MCAA exhibited a correlation with the clivus angle, suggesting that it may also serve as an indicator of foramen magnum width and, indirectly, skull base dysplasia.

The findings of the current study also showed that CCOS scores were closely related with the preoperative morphometric measurements of the craniovertebral junction, with MCAA, which assesses both osseous and soft tissues simultaneously, being independently associated with CCOS score. Nagashima et al., reported that syringomyelia failed to regress when the MCAA angle was <130° (25); however, in the current study, stratification of patients by CCOS scores showed that the cut-off MCAA value for poorer outcomes was 126°. This was in agreement with He et al., who reported a similar value of 127° in neutral position (11). These findings suggest that posterior decompression would provide insufficient relief in the craniovertebral junction in patients with MCAA values below the cut-off point. This, in turn, would result in unsatisfactory clinical outcomes and decreased shrinkage of syringomyelia.

Available evidence on the optimal surgical treatment measure for CM1 is contradictory, with various techniques such as craniectomy, craniectomy with duraplasty, and tonsil resection being suggested for posterior fossa decompression. Although Oliveira et al., reported no significant differences between these techniques, the current study showed that patient outcomes were dependent on their MCAA values (26). Patients exhibiting severe VCMC (MCAA < 126°) appeared to benefit most from CTR, although the overall general clinical outcomes of the patients were not optimal. This highlights the importance of evaluating ventral compression using MCAA when developing a surgical plan to ensure superior treatment outcomes.

This study had several limitations including its retrospective study design and small sample of patients. Another key limitation was that patients with severe ventral compression and low CCOS scores were yet to undergo a second surgery and, therefore, were still being followed-up. This prevented complete assessment of the effects of anterior decompression and risk of complications in this group. Future studies should use a prospective study design and a larger sample of patients with complete follow-up to provide more accurate results.

## Conclusion

The findings of this study suggest that preoperative assessment of MCAA in patients with CM1can contribute to development of an appropriate surgical treatment plan and, consequently, improve long-term patient health. Patients with larger MCAAs can be treated using less invasive methods, and the cut-off values of 126˚ and 138˚ may be used to assess surgical risk and benefit. Furthermore, anterior decompression should also be taken into consideration as a rescue strategy in case of failure of posterior approaches.

## Data Availability

The raw data supporting the conclusions of this article will be made available by the authors, without undue reservation.
